# The H-reflex study of the flexor carpi radialis muscle in healthy individuals

**DOI:** 10.3389/fneur.2024.1462882

**Published:** 2024-11-19

**Authors:** Metin Mercan, Reha Kuruoğlu

**Affiliations:** ^1^Department of Neurology, Bakirkoy Dr. Sadi Konuk Training and Research Hospital, Istanbul, Türkiye; ^2^Department of Neurology, Gazi University Faculty of Medicine, Ankara, Türkiye

**Keywords:** H-reflex, flexor carpi radialis, H-reflex latency, Hmax amplitude, Hmax/Mmax ratio

## Abstract

**Objective:**

This study aimed to investigate the physiological and anatomical factors influencing the flexor carpi radialis (FCR) H-reflex and to establish reference values for FCR H-reflex parameters in relation to these factors.

**Methods:**

The FCR H-reflexes, elicited by median nerve stimulation, were assessed in 80 healthy individuals both at rest and during isometric voluntary contraction (IVC). Multiple linear regression analyses were performed with H-reflex parameters as the dependent variables, while age, gender, height, arm length, and weight were included as independent variables.

**Results:**

The FCR H-reflex was recorded bilaterally in nearly all healthy individuals (76 out of 80) during IVC, while it could be obtained in only 35% (28 out of 80) of these individuals at rest. During IVC, the maximum H-reflex amplitude (Hmax) and its ratio to the maximum M-response amplitude (Hmax/Mmax ratio) were significantly increased (*p* < 0.001). However, there were no changes in H-reflex latency, latency difference, conduction velocity (HRCV), or amplitude ratio (*p* > 0.05). In both conditions, age and arm length were the most important factors affecting H-reflex latency (*p* < 0.001), while HRCV was influenced only by age (*p* < 0.01). Women exhibited shorter H-reflex latencies (*p* < 0.01), and both Hmax amplitude and Hmax/Mmax ratio were higher in women during IVC (*p* < 0.05). The H-reflex amplitude ratio during IVC showed a tendency to decrease with age (*p* < 0.05).

**Conclusion:**

These findings suggest that FCR H-reflexes are more reliably elicited during IVC, and that both physiological and anatomical factors should be considered when assessing H-reflex abnormalities.

## Introduction

The H-reflex is an effective tool for examining damage to proximal segments of the peripheral nerve and for measuring excitability changes in the motor neuron pool. It can be elicited in most muscles whose peripheral nerves are accessible through percutaneous electrical stimulation (1, 2). The generation of H-reflex depends on the monosynaptic excitation of alpha motor neurons by group Ia sensory afferents, but it is modulated through both segmental and supraspinal pathways. For instance, converging excitatory postsynaptic inputs bring a larger number of motoneurons closer to their firing threshold, thereby resulting in a notable increase in the magnitude of the H-reflex (2, 3). Additionally, the strongest monosynaptic connections from group Ia sensory afferents are found in alpha motor neurons that innervate antigravity muscles (3, 4). As a result, the H-reflex can be readily elicited in antigravity muscles even at rest, whereas it rarely occurs in many flexor muscles. However, in both clinical and research settings, the flexor carpi radialis (FCR) muscle has been one of the most frequently studied, alongside the calf muscles (1, 2, 4).

To date, numerous H-reflex parameters have been described for use in clinical studies. Among these, the latency and velocity of the H-reflex reflect on the conduction properties of group Ia sensory afferents and alpha motor neurons, as well as the synaptic delay (1, 5). The maximum amplitude of the H-reflex (Hmax) and its ratio to the maximum amplitude of M-response (Hmax/Mmax) are associated with the number of alpha motor neurons activated by group Ia sensory afferents, depending on the net influence of presynaptic and postsynaptic projections within the reflex arc (2, 5). Common H-reflex abnormalities in peripheral nerve diseases include prolonged onset latency, absence of the H-reflex on the affected side, and increased latency difference between the right and left sides (6). Furthermore, many researchers agree that H-reflex recordings are superior to electromyography studies in detecting mild or early radiculopathy (7, 8). However, the sensitivity of FCR H-reflex studies has been reported to vary widely, ranging from 3.7 to 50% in C6 radiculopathy and from 33 to 88% in C7 radiculopathy (7, 9–12). This variability is likely due to differences in patient samples, criteria for describing abnormal H-reflexes, recording techniques, and the validity of normative values (7, 8, 11–13). In H-reflex recordings on patients with pyramidal signs, the Hmax amplitude and Hmax/Mmax ratio have been shown to be significantly higher than healthy subjects (14). In contrast, these values diminish greatly due to axonal loss in patients with polyneuropathy or radiculopathy (10, 12, 15, 16).

It is well known that isometric voluntary contraction (IVC) strongly increases the size of monosynaptic reflexes (1, 4, 14). In daily electrodiagnostic practice, particularly for upper extremity muscles, when the reflex response cannot be evoked by electrical stimulation, mild voluntary contraction of either remote muscles or the muscles under examination is routinely used as a facilitation technique. However, due to the nature of reflex responses, results obtained at rest and during IVC may differ, potentially complicating the interpretation of H-reflex parameters (1–5). Most studies on H-reflex normative data have not distinguished between these two conditions. Additionally, these parameters may be influenced by factors such as age, gender, and height (6). In consideration of these shortcomings, we aimed to establish normative values for FCR H-reflex parameters and to assess the effects of physiological and anatomical factors under both conditions, for use in routine electrophysiological recordings.

## Materials and methods

### Participants

The study population consisted of healthy individuals with normal conduction studies for the median and ulnar nerves, as well as normal physical examinations. Age, gender, height, arm length, and weight were recorded for all participants. Individuals were excluded if they had a history of neurological or systemic disease, reported any signs or symptoms of a neuromuscular disorder, or used medications that might affect spinal excitability or nerve conduction studies. This study was approved by the Ethics Committee of Gazi University Faculty of Medicine (protocol number: 148). Written informed consent was obtained from all participants.

### Electrophysiological studies

**Figure 1:**
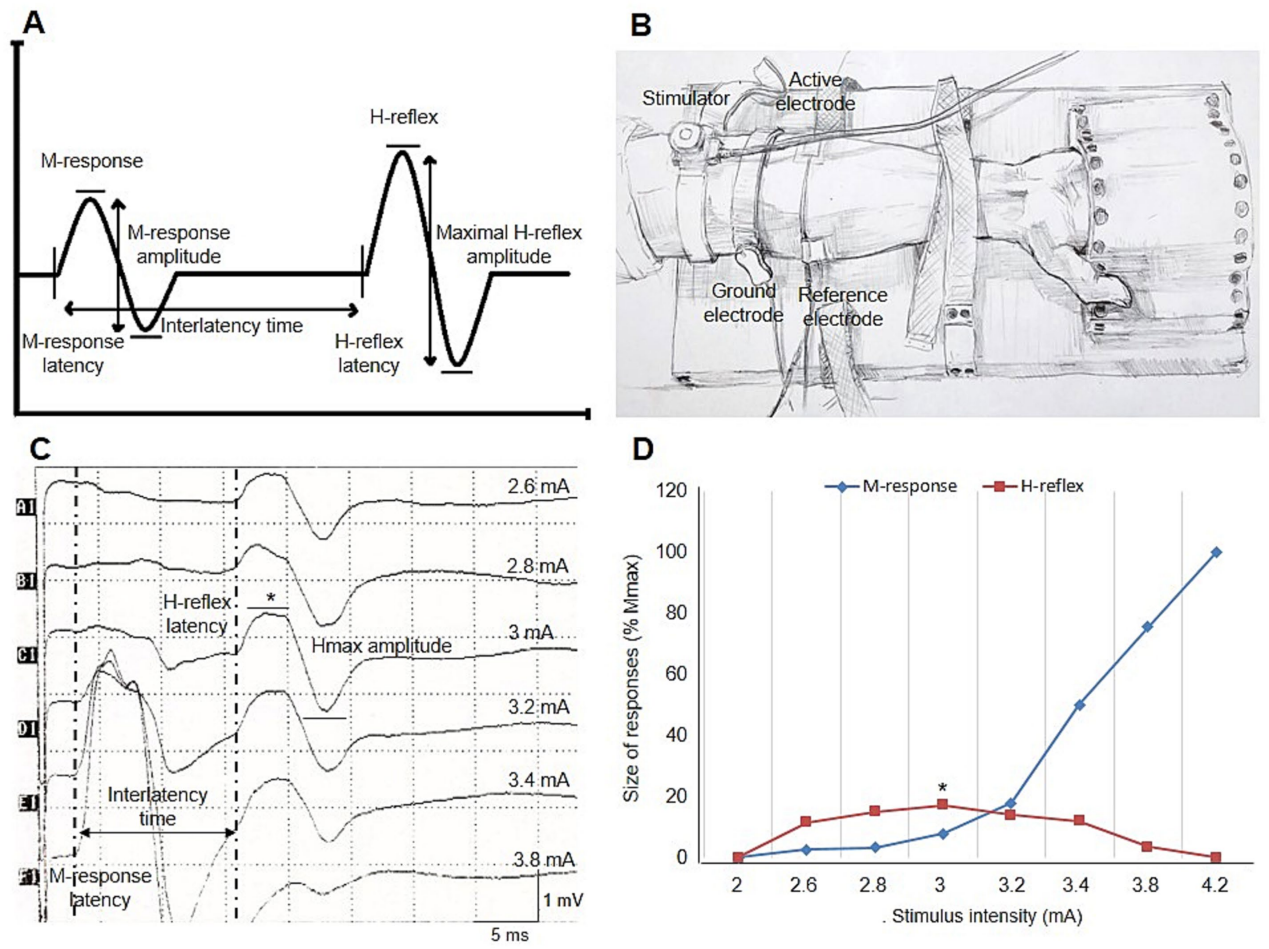
(A) Illustration of H-reflex measurements. (B) Placement of electrodes and percutaneous stimulator for recording the FCR H-reflex in the supine wrist position. (C) Right FCR H-reflex recording in a healthy individual at rest. Sample measurements for latency, peak-to-peak amplitude, and interlatency time of the H-reflex and M-response are shown [H-reflex latency: 15.6 ms, Hmax amplitude: 1.7 mV, M-response latency: 2.9 ms, interlatency time: 12.7 ms, and HRCV: 2 × 440 mm/(12.7 ms – 1 ms) = 75.2 m/s]. (D) Typical example of the recruitment curve for the H-reflex and M-responses in the same trace. In both curves, the H-reflex and M-response are presented as a percentage of maximal M-response. Mmax amplitude and the Hmax/Mmax ratio are 9.8 mV and 17.3%, respectively. Asterisks indicate the maximal H-reflex in this healthy individual (C,D). FCR, flexor carpi radialis; Hmax, maximal H-reflex amplitude; HRCV, H-reflex conduction velocity; Mmax, maximal M-response amplitude.

The FCR H-reflex studies were conducted using Ag-AgCl surface recording electrodes with a Neuropack *Σ* MEB-5504 K electromyograph (Nihon Kohden Corp., Tokyo, Japan). H-reflex recordings of the FCR muscle were obtained in a quiet room with the individuals lying comfortably in supine position, following the method previously described by Jabre (17). The H-reflex parameters, particularly the amplitude, can vary significantly depending on the position of the forearm. Therefore, the hand and forearm on the examined side were immobilized with a heavy board; the hand was secured in a receptacle made of hard material, and the forearm was firmly fixed to the board with velcro strips, which were also helpful in maintaining a constant IVC during wrist flexion (Figure 1B). All individuals were tested bilaterally under two different conditions: at rest and during IVC. Individuals were instructed to maintain an IVC of 10 to 30% of their predetermined maximum voluntary contraction (MVC), monitored by auditory feedback. A minimum of 1 min was allowed between rest and IVC sets to prevent reflex attenuation. The skin temperature of the examined upper limb ranged from 31 to 35°C.

Recordings were obtained with a sweep speed of 5 ms/div, a sensitivity of 0.5–1 mV/div, a sampling frequency of 20,000 Hz, and a bandpass filter of 20–10,000 Hz. Stimuli consisted of 0.5-ms duration pulses, which were sufficiently long to preferentially recruit group Ia afferent fibers. A single submaximal stimulus, delivered no more than once every 7–10 s at an irregular rate, was applied to the median nerve at the antecubital fossa using a bipolar stimulating probe, with the cathode placed proximal to the anode. Stimulus intensity typically started at 1 mA and was increased in 0.2–0.4 mA increments until a maximum M response was achieved. To obtain satisfactory recordings, especially during IVC, stimuli were often delivered 2–3 times at the intensity that produced the largest reflex response. The largest potentials with stable onset latency and shape, clearly separated from background noise, were accepted for data analysis. All measured responses exhibited the characteristic features of the H-reflex (1, 2, 5).

The H-reflex latency was measured to the onset of the first negative deflection from the baseline (Figure 1A). The difference in onset latency between the right and left sides was defined as the H-reflex latency difference. The Hmax amplitude was measured from peak to peak (Figure 1A) and also expressed as a percentage of the highest M-response amplitude (Hmax/Mmax ratio). The H-reflex amplitude ratio was calculated by dividing the smaller Hmax amplitude by the larger Hmax amplitude, reflecting the symmetry of Hmax amplitude between the right and left sides. Arm length was measured from the medial epicondyle to the C6 spinous process using a caliper, with the arm pronated on the coronal plane and abducted at a 90-degree angle. The H-reflex conduction velocity (HRCV) was calculated using the following formula, as described in previous reports (18):

$\mathrm{HRCV}\;\left(m/s\right)=2\;\mathrm{xarm length}\;\left(\mathrm{mm}\right)/\left(\mathrm{interlatency time}\;\left(\mathrm{ms}\right)-1\;\mathrm{ms}\right)$

The interlatency time was calculated by subtracting the M-response latency from the H-reflex latency. The monosynaptic delay, estimated to be approximately 1 ms, was included in the formula. H-reflex parameters and the experimental setup for recording the FCR H-reflex are illustrated in Figure 1.

### Statistical analysis

The descriptive characteristics were expressed as mean ± standard deviation (SD) for continuous variables, and as numbers and percentages for categorical variables. The continuous variables were tested for normality with the Shapiro–Wilk and Kolmogorov–Smirnov tests. Differences in the values of continuous variables were compared by the Student’s *t*-test or the Mann–Whitney U test according to their distribution. Pearson’s or Spearman’s correlation coefficients were used to assess the associations between H-reflex parameters and physiological or anatomical factors. Furthermore, to build the predictive models between each normally-distributed H-reflex parameter obtained at rest and during IVC and factors such as age, gender, height, arm length, and weight, multiple regression analysis was performed using the stepwise method. Significance thresholds were set at ≤0.05 for a predictor to enter the model and at ≥0.10 for it to be removed. The R^2^ and adjusted R^2^ statistics indicated how much of the variability in the H-reflex parameters is explained by the regression models. Findings were considered statistically significant when *p* < 0.05. Upper and lower limits for H-reflex parameters were calculated by adding or subtracting 2 SD from the mean values. For non-normally distributed data, the 97.5th and 2.5th percentiles were used.

## Results

### Demographic data

Eighty healthy individuals with a mean± SD age of 45.1 ± 15.5 years (range: 20–75 years) were assessed. There were 39 men (mean ± SD: 44.2 ± 16.5 years) and 41 women (mean ± SD: 45.8 ± 14.6 years). The age was not statistically different between genders (*p* = 0.659). However, arm length, height, and weight were significantly different between men and women: 45.4 ± 2.4 cm vs. 42.4 ± 1.9 cm (*p* < 0.001), 173.1 ± 7.6 cm vs. 161 ± 5.6 cm (*p* < 0.001), and 77.4 ± 12 kg vs. 70.5 ± 11.6 kg (*p* = 0.013), respectively.

### Influences of IVC on FCR H-reflex parameters

The FCR H-reflex was obtained bilaterally in only 28 (35%) healthy individuals at rest, whereas it was recordable bilaterally in 76 (95%) during IVC. The FCR H-reflex was elicited unilaterally in 3 (3.8%) and 1 (1.3%) healthy individuals at rest and during IVC, respectively. There were no significant differences between measurements recorded at rest and during IVC in terms of H-reflex latency, latency difference, amplitude ratio, and HRCV (*p* > 0.05). However, significantly higher values were observed for Hmax amplitude and Hmax/Mmax ratio during IVC compared to those recorded at rest (*p* < 0.001) (Table 1).

**Table 1:** Comparison of H-reflex parameters obtained during IVC and at rest.

Parameters	Right			Left		
	Rest mean ± SD (*n* = 30)	IVC mean ± SD (*n* = 31)	*p*-value	Rest mean ± SD (*n* = 29)	IVC mean ± SD (*n* = 30)	*p*-value
H-reflex latency (ms)	15.6 ± 1.05	15.5 ± 0.95	0.915	15.5 ± 1.11	15.5 ± 1.06	0.785
H-reflex latency difference (ms)				0.20 ± 0.16	0.25 ± 0.17	0.253*
HRCV (m/s)	74.9 ± 4.41	75 ± 4.14	0.989	74.9 ± 4.73	75.4 ± 4.83	0.696
Hmax amplitude (mV)	1.70 ± 0.94	2.77 ± 1.14	< 0.001*	1.47 ± 1.07	2.38 ± 0.94	<0.001*
Hmax/Mmax ratio (%)	18.1 ± 11.0	29.3 ± 13.2	< 0.001*	15.9 ± 12.5	25.6 ± 11.3	<0.001*
H-reflex amplitude ratio				0.66 ± 0.241	0.75 ± 0.157	0.087

Hmax, maximal H-reflex amplitude; HRCV, H-reflex conduction velocity; IVC, isometric voluntary contraction; Mmax, maximal M-response amplitude; *n*, number of individuals; SD, standard deviation. The H-reflex latency difference was calculated by subtracting the shorter latency from the longer. The H-reflex amplitude ratio was derived by dividing the smaller amplitude by the larger. For this reason, only one value appears in the table for these two parameters at IVC and at rest, and they are compared between the two conditions. The Student’s *t*-test was applied for two-group comparisons. *Non-parametric Mann–Whitney U tests were used to assess the differences. *p* values demonstrating statistical significance are displayed in bold numbers.

### Interrelationship between physiological and anatomical factors and FCR H-reflex parameters

When H-reflex parameters were compared between genders, H-reflex latency was significantly longer in men than in women both at rest and during IVC (*p* < 0.01). Additionally, during IVC, women had higher Hmax amplitude and Hmax/Mmax ratio than men (*p* < 0.05), while no significant differences were observed at rest (*p* > 0.05, Table 2).

**Table 2:** Comparison of H-reflex parameters between men and women.

Parameters	Rest			IVC		
	Men mean ± SD (R: *n* = 11) (L: *n* = 10)	Women mean ± SD (R: *n* = 19) (L: *n* = 19)	*p*-value	Men mean ± SD (R: *n* = 36) (L: *n* = 35)	Women mean ± SD (R: *n* = 41) (L: *n* = 41)	*p*-value
R H-reflex latency (ms)	16.3 ± 0.90	15.2 ± 0.92	**0.004**	16.5 ± 0.88	15.2 ± 0.84	**<0.001**
L H-reflex latency (ms)	16.4 ± 0.83	15.1 ± 0.96	**<0.001**	16.5 ± 0.96	15.2 ± 0.86	**<0.001**
H-reflex latency difference (ms)	0.17 ± 0.12	0.22 ± 0.18	0.562*	0.27 ± 0.17	0.26 ± 0.18	0.803*
R HRCV (m/s)	74.9 ± 5.40	75.0 ± 3.89	0.953	72.9 ± 4.59	74.2 ± 4.41	0.217
L HRCV (m/s)	75.6 ± 5.09	74.6 ± 4.63	0.591	73.2 ± 5.17	74.4 ± 4.65	0.266
R Hmax amplitude (mV)	1.60 ± 0.97	1.75 ± 0.95	0.914*	2.08 ± 1.03	2.57 ± 1.05	0.013*
L Hmax amplitude (mV)	1.30 ± 1.10	1.56 ± 1.07	0.261*	1.84 ± 0.97	2.35 ± 0.88	0.009*
R Hmax/Mmax ratio (%)	15.4 ± 10.7	19.7 ± 11.1	0.355*	19.1 ± 10.1	29.6 ± 10.9	**<0.001**
L Hmax/Mmax ratio (%)	11.5 ± 7.7	18.2 ± 14.1	0.155*	17.4 ± 9.0	27.6 ± 10.5	**<0.001**
H-reflex amplitude ratio	0.68 ± 0.244	0.64 ± 0.246	0.691	0.72 ± 0.165	0.75 ± 0.160	0.359

Hmax, maximal H-reflex amplitude; HRCV, H-reflex conduction velocity; IVC, isometric voluntary contraction; L, left; Mmax, maximal M-response amplitude; *n*, number of individuals; R, right; SD, standard deviation. The Student’s *t*-test was applied for two-group comparisons. *Non-parametric Mann–Whitney U tests were used to assess the differences. *p* values demonstrating statistical significance are displayed in bold numbers.

All H-reflex parameters were normally distributed, except for H-reflex latency difference and Hmax amplitude. Therefore, predictive regression models could be created for H-reflex latency, HRCV, Hmax/Mmax ratio, and H-reflex amplitude ratio. The descriptive data and regression models for FCR H-reflex parameters are listed in Tables 3, 4. During IVC, H-reflex latency difference was significantly and positively correlated with age (rho = 0.310, *p* = 0.006); however, a model could not be formed because the data did not fit a normal distribution. Correlation analyses did not reveal any significant relationship between Hmax amplitude, H-reflex amplitude ratio, or Hmax/Mmax ratio at rest and any of the physiological and anatomical factors (Table 5).

**Table 3:** The normative data and regression models for H-reflex parameters at rest.

Parameters	*n*	Mean ± SD	Normative value	Regression model	R^2^	*p*-value
R H-reflex latency (ms)						
Total	30	15.6 ± 1.05	<17.7	1.156 + 0.302 x arm lenght (cm) + 0.027 x age (years)	0.609**	**<0.001**
Women	19	15.2 ± 0.92	<17.0			
Men	11	16.3 ± 0.90	<18.1			
L H-reflex latency (ms)						
Total	29	15.5 ± 1.11	<17.7	0.566 + 0.308 x arm lenght (cm) + 0.033 x age (years)	0.661**	**<0.001**
Women	19	15.1 ± 0.96	<17.0			
Men	10	16.4 ± 0.83	<18.1			
H-reflex latency difference (ms)	28	0.20 ± 0.16	-	NA		
R HRCV (m/s)	30	74.9 ± 4.41	>66.1	82.311–0.178 x age (years)	0.256	**0.004**
L HRCV (m/s)	29	74.9 ± 4.73	>65.4	84.189–0.227 x age (years)	0.410	**<0.001**
R Hmax amplitude (mV)	30	1.70 ± 0.94	>0.66*	NA		
L Hmax amplitude (mV)	29	1.47 ± 1.07	>0.32*	NA		
R Hmax/Mmax ratio (%)	30	18.1 ± 11.0	>4.30*	NA		
L Hmax/Mmax ratio (%)	29	15.9 ± 12.5	>0.96*	NA		
H-reflex amplitude ratio	28	0.66 ± 0.241	0.178–1	NA		

Hmax, maximal H-reflex amplitude; HRCV, H-reflex conduction velocity; L, left; Mmax, maximal M-response amplitude; NA, not applicable; *n*, number of individuals; R, right; R^2^, coefficient of determination; SD, standard deviation. *Non-normally distributed data, **Given as adjusted R^2^. Range is given for the H-reflex amplitude ratio. *p* values demonstrating statistical significance are displayed in bold numbers.

**Table 4:** The normative data and regression models for H-reflex parameters during IVC.

Parameters	*n*	Mean ± SD	Normative value	Regression model	R^2^	*p*-value
R H-reflex latency (ms)						
Total	77	15.8 ± 1.05	<17.9	1.099 + 0.302 x arm lenght (cm) + 0.033 x age (years)	0.652**	**<0.001**
Women	41	15.2 ± 0.84	<16.9			
Men	36	16.5 ± 0.88	<18.3			
L H-reflex latency (ms)						
Total	76	15.8 ± 1.12	<18.0	−0.113 + 0.323 x arm lenght (cm) + 0.038 x age (years)	0.687**	**<0.001**
Women	41	15.2 ± 0.86	<16.9			
Men	35	16.5 ± 0.96	<18.4			
H-reflex latency difference (ms)	76	0.26 ± 0.18	<0.70*	NA		
R HRCV (m/s)	77	73.6 ± 4.51	>64.6	81.157–0.168 x age (years)	0.332	**<0.001**
L HRCV (m/s)	76	73.8 ± 4.90	>64.0	82.762–0.197 x age (years)	0.389	**<0.001**
R Hmax amplitude (mV)						
Total	77	2.34 ± 1.06	1–4.77*	NA		
Women	41	2.57 ± 1.05	1.13–4.73*			
Men	36	2.08 ± 1.03	>0.93*			
L Hmax amplitude (mV)						
Total	76	2.11 ± 0.95	0.62–4.30*	NA		
Women	41	2.35 ± 0.88	0.79–4.57*			
Men	35	1.84 ± 0.97	>0.60*			
R Hmax/Mmax ratio (%)						
Total	77	24.7 ± 11.7	1.3–48.1	19.071 + 10.546 x gender	0.205	**<0.001**
Women	41	29.6 ± 10.9	7.8–51.4			
Men	36	19.1 ± 10.1	<39.3			
L Hmax/Mmax ratio (%)						
Total	76	22.9 ± 11.1	0.7–45.1	17.349 + 10.212 x gender	0.215	**<0.001**
Women	41	27.6 ± 10.5	6.6–48.6			
Men	35	17.4 ± 9.0	<35.4			
H-reflex amplitude ratio	76	0.74 ± 0.162	0.416–1	0.86–0.003 x age (years)	0.066	**0.025**

Hmax, maximal H-reflex amplitude; HRCV, H-reflex conduction velocity; L, left; Mmax, maximal M-response amplitude; NA, not applicable; *n*, number of individuals; R, right; R^2^, coefficient of determination; SD, standard deviation. *Non-normally distributed data, **Given as adjusted R^2^. Range is given for the Hmax amplitude, Hmax/Mmax ratio and H-reflex amplitude ratio. *p* values demonstrating statistical significance are displayed in bold numbers. Women and men were coded 1 and 0 respectively, in the regression equation for Hmax/Mmax amplitude ratios.

**Table 5:** Correlation between physiological and anatomical factors and H-reflex parameters.

	Correlation coefficient									
	Age (years)		Gender (W/M)		Height (cm)		Arm length (cm)		Weight (Kg)	
	Rest	IVC	Rest	IVC	Rest	IVC	Rest	IVC	Rest	IVC
R H-reflex latency (ms)	0.156	0.350**	−0.512**	−0.591**	0.484**	0.526**	0.732**	0.656**	0.447**	0.472**
L H-reflex latency (ms)	0.130	0.380**	−0.591**	−0.592**	0.557**	0.497**	0.737**	0.654**	0.543**	0.507**
H-reflex latency difference (ms)	−0.172	0.310**	−0.112	−0.029	−0.010	−0.088	−0.139	−0.190	−0.163	0.019
R HRCV (m/s)	−0.506**	−0.557**	−0.110	0.142	−0.11	0.018	−0.015	0.075	−0.106	−0.220*
L HRCV (m/s)	−0.640**	−0.624**	0.104	0.129	0.208	0.085	0.084	0.101	−0.181	−0.264*
R Hmax amplitude (mV)	−0.070	−0.020	−0.020	0.283*	0.023	−0,143	0.173	0.056	0.041	−0,071
L Hmax amplitude (mV)	−0.076	−0.057	−0.212	0.300**	−0.078	−0.183	0.001	−0.082	−0.267	−0.078
R Hmax/Mmax ratio (%)	−0.038	0.006	−0.172	0.452**	−0.113	−0.268*	0.032	−0.131	−0.013	−0.107
L Hmax/Mmax ratio (%)	0.032	0.053	−0.269	0.464**	−0.161	−0.454**	−0.036	−0.272**	−0.182	−0.182
H-reflex amplitude ratio	−0.121	−0.257*	0.079	0.107	−0.047	−0.007	−0.154	0.063	−0.217	−0.072

Hmax, maximal H-reflex amplitude; HRCV, H-reflex conduction velocity; IVC, isometric voluntary contraction; L, left; M, men; Mmax, maximal M-response amplitude; R, right; SD, standard deviation; W, women. *Correlation is significant at the < 0.05 level; ** Correlation is significant at the < 0.01 level. Significant correlations are displayed in bold numbers.

### H-reflex latency

**Figure 2:**
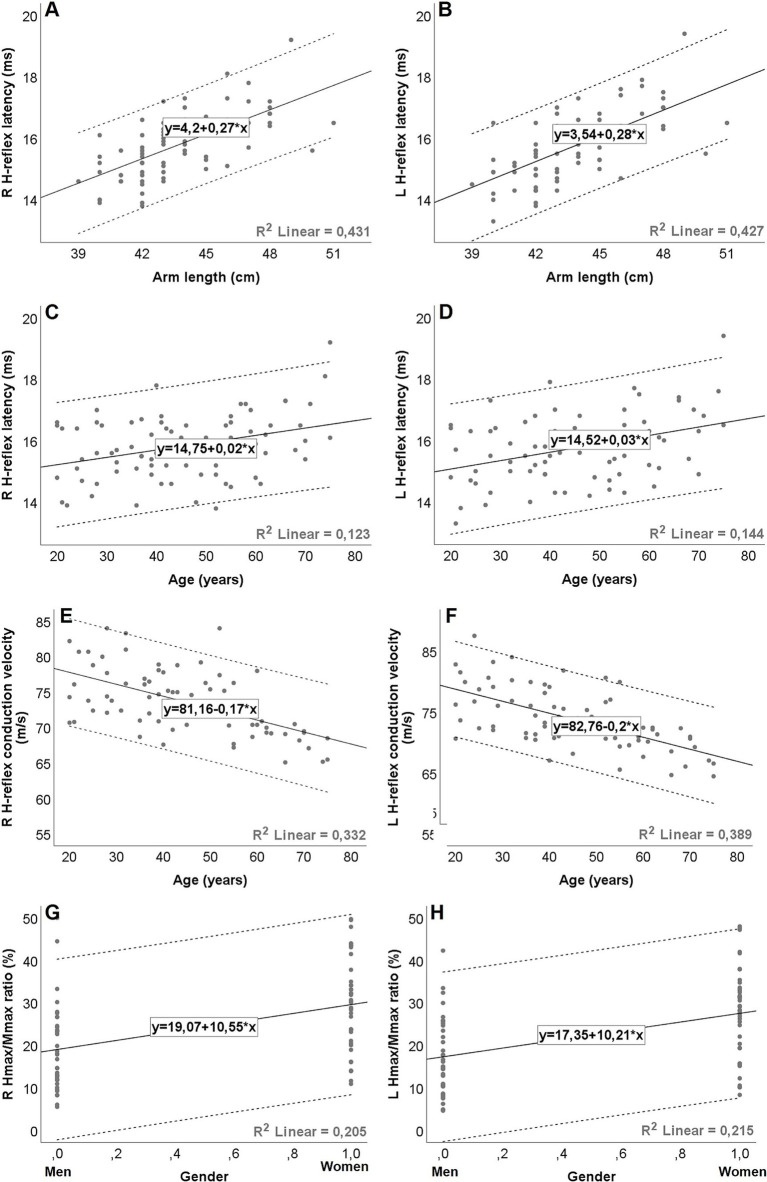
Graphical representation (A–H) of simple linear regression equations for H-reflex parameters during isometric voluntary contraction, with dashed lines representing the 95% prediction intervals (ie, the statistical range for which there is a 95% probability that subsequent values will be within the range). The expected value is calculated as follows: H-reflex parameters = Constant + (slope/coefficient) * factor. Gender coded as men = 0; women = 1. R^2^ = coefficient of determination. Hmax, maximal H-reflex amplitude; Mmax, maximal M-response amplitude; R, right; L, left.

At both rest and during IVC, H-reflex latency was positively correlated with height, arm length, and weight. A significant positive correlation with age was also found during IVC. Furthermore, a moderate association with gender was noted under both conditions (Table 5). The multiple regression analysis showed that arm length, age, and gender were the factors that entered the model during IVC (*p* < 0.001); however, gender did not have a significant impact at rest (*p* > 0.05). Among the associated factors, arm length showed the strongest correlation with H-reflex latency both at rest and during IVC, explaining almost half of the latency variability, with the adjusted R^2^ ranging from 0.42 to 0.53 (*p* < 0.001). When age was included into the final model, the adjusted R^2^ increased to 0.61–0.69, indicating that age explains an additional 9–26.7% of the variability in latency (*p* < 0.001). On the other hand, during IVC, the regression model demonstrated a tendency for higher adjusted R^2^ values (Right: 0.69, Left: 0.72) when gender was added, compared to the model with only two variables (age and arm length). However, gender only explained an additional 3–4% of the variability in models with three variables (Right: *p* = 0.002, Left: *p* = 0.003). The results of the regression analyses and the corresponding regression models are shown in Tables 3, 4, as well as in Figures 2A–D.

### H-reflex conduction velocity

HRCV was inversely correlated with age both at rest and during IVC. A negative correlation with weight was also observed, but only during IVC (Table 5). However, in the multiple regression analysis, HRCV was significantly influenced only by age in both conditions (*p* < 0.001; Tables 3, 4; Figures 2E,F). The regression models accounted for approximately 25.6–41% of the variability (*p* < 0.001).

### Hmax/Mmax ratio

The Hmax/Mmax ratio showed a significant association with gender, arm length, and height during IVC, but not at rest (Table 5). However, multiple regression analysis demonstrated that only gender had a significant impact on the Hmax/Mmax ratio (*p* < 0.001, Table 4). With gender in the model, the R^2^ for the Hmax/Mmax ratio was 0.21 on the right side and 0.22 on the left side (*p* < 0.001, Figures 2G,H).

### H-reflex amplitude ratio

A weak negative correlation was found between age and the H-reflex amplitude ratio during IVC (*r* = −0.257, *p* = 0.012), although it was quite limited, as the regression model explained only 6.6% of the variability (*p* = 0.025, Table 4).

## Discussion

The results of previous studies investigating the elicitation of the FCR H-reflex have shown considerable variability, particularly under resting conditions. Reflex responses were reportedly obtained in 28.5–95% of healthy individuals, while recordings were successful in 90–100% of cases with the use of facilitation maneuvers (11, 12, 18–21). In our experience, the FCR H-reflex was unobtainable at rest using surface electrodes in the majority of healthy individuals, and IVC was often required to evoke the reflex response, which also increased the Hmax amplitude and Hmax/Mmax ratio. Furthermore, our study revealed that age, gender, and arm length significantly influence FCR H-reflex parameters. However, the extent of the effects of these physiological and anatomical factors was not identical for each parameter under different conditions. H-reflex latency was prolonged both at rest and during IVC with increasing arm length and age, while HRCV slowed in both conditions as individuals aged. The H-reflex latency difference and amplitude ratios showed weak correlations with age, but only during IVC. Women exhibited higher H-reflex amplitudes and Hmax/Mmax ratios during IVC compared to men.

Numerous studies have reported reference values for FCR H-reflex parameters, though the results are inconsistent, likely due to the different methodologies employed. Normative values for the upper limit of latency range from 17 to 19 ms (17, 20, 21). However, multiple regression analyses show that latency depends on physiological and anatomical factors (6). The regression equations established by Schimsheimer et al. have been cited in many electrodiagnostic texts (1, 16, 22). The authors maintain that arm length and height have similar predictive value for latency, with age having little impact and no relationship to gender. They concluded that reference values based on height or arm length alone could be used in the clinical setting, as these factors explain the majority of the variability (11, 12, 16). Some studies have supported these findings by noting no significant association between age and latency (21, 23). The widely proposed multiple regression formula for improving the accuracy of FCR H-reflex latency is as follows: H-reflex latency (ms) = −0.44 + 0.0925 x Height (cm) + 0.0316 x Age (years) ± 0.83 (SD) (16). Men tend to have slightly prolonged latency compared to women (13), likely due to larger body size, which may explain why the gender variable is often excluded from regression equations (23, 24). On the other hand, Huang et al. reported a significant relationship between latency and gender (25), which led us to perform a stepwise linear regression analysis including gender as an independent variable. R^2^ statistics demonstrated that arm length and age explained 42–53% and 12–14% of the variability in H-reflex latency, respectively. The effect of gender was significant only during IVC. When gender was the only independent variable in the regression model, it explained 35% of the variability. However, the additional effect of gender on H-reflex latency in the model with three variables was minimal (3–4%), with the majority of the latency variability between genders actually attributable to arm length. As a result, we opted for a regression model that takes into account only age and arm length variables.

In clinical practice, side-to-side comparisons of H-reflex latency are useful for determining the presence of unilateral nerve or root damage (6–12, 21, 22). Although the effects of physiological and anatomical factors on this measure had not been investigated in detail previously, our findings revealed no associations at rest or during IVC. Additionally, in previous studies on non-normally distributed data, reference values were derived using the mean ± some number of SD or the highest difference, resulting in a wide range of normative data from 0.6 to 2.4 ms (13, 17, 21, 26). We employed a more accurate statistical approach, defining normal values by the 2.5th and 97.5th percentiles. Our analysis revealed that the H-reflex latency difference should not exceed 0.7 ms.

Most researchers agree on the negative effect of age on motor and sensory nerve conduction velocity (NCV) (27, 28). However, the associations of NCV with height reported in the literature are less consistent than those with latency (29–31). Moreover, this association differs between the nerves of the upper and lower limbs. In taller individuals, it has been suggested that NCV is slower in the lower limbs due to a length-dependent reduction in fiber diameter distally. Additionally, taller individuals may have longer nodes of Ranvier than shorter individuals, which could slow saltatory conduction across the nodes of Ranvier (29, 32). Research on the influence of gender on NCV has yielded contradictory results (30). Some electrophysiological studies have found that women have faster motor or sensory NCV compared to men, while others have not established this difference. The discrepancy between genders is often attributed to the influence of limb length rather than sex-specific variations in the peripheral nervous system (28–33). To our knowledge, normative data on FCR HRCV have been reported in only one study (mean ± SD = 73.7 ± 7.2 m/s, cutoff point 59.3 m/s) (18), which demonstrated a negative effect of age but no difference between genders. Similarly, our findings using regression models revealed a negative association with age both at rest and during IVC, explaining a notable portion of the variability in HRCV. This study also determined the normal ranges for HRCV and plotted the 95% confidence limits for HRCV against age using regression models.

The Hmax amplitude and Hmax/Mmax ratio are influenced by several factors, including postural variation, joint angle, muscle stretch, remote muscle activity, and mental effort (1, 2, 4–6). Studies have demonstrated that the H-reflex is suppressed by passive muscle lengthening and antagonist muscle activity due to presynaptic inhibition of Ia terminals (2, 34, 35). Additionally, changes in wrist position can significantly affect the generation of the H-reflex through presynaptic inputs to Ia afferents terminals (36–39). Furthermore, variations in H-reflex magnitude are closely related to the level of ongoing EMG activity in the test muscle (2, 4, 6, 26). Therefore, Hmax amplitude is not commonly utilized in clinical practice for diagnosing neuromuscular disorders. However, despite variability in the Hmax/Mmax ratio and the H-reflex amplitude ratio, the reported reference values for these parameters generally remain within acceptable limits (1, 6, 7, 22, 40). Clinical studies have identified an H-reflex amplitude ratio of 0.4 or higher in healthy individuals (7, 40, 41). The normal Hmax/Mmax ratio has frequently been reported to range between 0.5 and 0.7, although it may vary depending on the specific muscles and facilitation maneuvers (6). In our study, these parameters were more reliably measured during IVC than at rest. This may be due to the maintained stability of motoneuron excitability during IVC (4, 5). The normative range of the Hmax/Mmax ratio and the H-reflex amplitude ratio during IVC was found to be between 0.7 and 51%, and 0.42 and 1, respectively. Associations between H-reflex magnitude measurements and factors such as age and gender have generally been explored in the lower limbs, with inconsistent results. It has been reported that both Hmax amplitude and the Hmax/Mmax ratio gradually decrease with age (42, 43). Similarly, the increase in H-reflex output in response to facilitation maneuvers has been suggested to diminish with age (44). However, some studies have demonstrated no difference in H-reflex magnitude between younger and older individuals (45–47). Consistent with these findings, our measures of Hmax amplitude and the Hmax/Mmax ratio did not show age-related changes in either condition, although the H-reflex amplitude ratio exhibited a negligible association with age during IVC. Similarly, a previous study also found no significant association between the H-reflex amplitude ratio and age (47). Interestingly, women had significantly higher Hmax amplitude and Hmax/Mmax ratio compared to men during IVC. Furthermore, our multiple linear regression models indicated that gender accounts for up to 22% of the variability in the Hmax/Mmax ratio. In a study on the soleus H-reflex, women were observed to have a lower Hmax/Mmax ratio compared to men (48). However, Hoffman et al. found no interaction between gender and the Hmax/Mmax ratio (49), and later reported a higher Hmax/Mmax ratio in women (50). The H-reflex magnitude primarily depends on the number of motor units activated by Ia afferents (4–6). Therefore, it may be hypothesized that the modulation of the FCR H-reflex during IVC differs between genders, probably due to differences in peripheral or supraspinal connectivity at the spinal level (51).

We did not account for the menstrual cycle when recording the Hmax/Mmax ratio and Hmax amplitude, which may be considered a limitation of this study. Sex hormone levels could potentially influence H-reflex amplitudes and Hmax/Mmax ratios (49, 50). Additionally, we did not measure IVC using a dynamometer or monitor background EMG activity via visual feedback. Relying solely on auditory feedback and verbal instructions may have been insufficient to maintain a constant muscle contraction of approximately 10–30% of MVC.

In conclusion, our findings revealed that physiological and anatomical factors should be considered when interpreting normal values of FCR H-reflex parameters for detecting abnormalities in clinical settings. The influence of each factor varies depending on the parameters tested. FCR H-reflex recordings obtained during IVC are likely to enhance the diagnostic utility of electrophysiological examinations. As a result, we present a dataset that can serve as a reference range for each of the FCR H-reflex parameters.

## Data Availability

The original contributions presented in the study are included in the article/supplementary material, further inquiries can be directed to the corresponding author.
